# Vaping in Asthmatic Adolescents: Time to Deal with the Elephant in the Room

**DOI:** 10.3390/children9030311

**Published:** 2022-02-24

**Authors:** Grigorios Chatziparasidis, Ahmad Kantar

**Affiliations:** 1 Primary Cilia Dyskinesia Unit, School of Medicine, University of Thessaly, 41110 Thessaly, Greece; 2 Pediatric Asthma and Cough Centre, Gruppo Ospedaliero San Donato, Bergamo and University Vita Salute San Raffaele, 24046 Milano, Italy; kantar@centropediatricotosse.com

**Keywords:** vaping, electronic cigarettes, smoking, asthma, adolescence

## Abstract

Adolescence is a period characterized by developmental, psychological, and psychosocial alterations, with a major impact on youths’ attitudes and perceptions. Adolescents with asthma may not comply with treatment and may develop risky behaviors, including smoking, vaping, and other substance use, leading to unanticipated exacerbations and consequences. Vaping has become extremely popular in this age group, and studies have suggested that it has potential adverse effects on asthmatic airways. More well-designed studies are needed to confirm the initial worrying data, and action must be taken by both medical officers and health authorities to deal with the elephant in the room and curb the vaping pandemic. The aim of this paper is to provide a review of the current knowledge regarding the effect of vaping on adolescents with asthma and to propose actions to restrain this fast-growing trend.

## 1. Introduction

The World Health Organization defines adolescence as a period of growth and development between puberty and adulthood, spanning across the ages of 10 to 19 years [1]. Asthma is common in adolescence, with 14.3% of 13–14-year-olds in Western Europe reporting a wheeze in the previous 12 months (current wheeze). The prevalence of more severe asthma in adolescents in Europe is estimated to be approximately 6.2% of all asthma [2]. Furthermore, a national health survey run by the Australian Institute of Health and Welfare in 2017–2018 found that the asthma prevalence in young people aged 15–24 years was around 10% in both sexes [3].

Contrary to what is commonly believed, asthma remission is not predicted by puberty, and nearly two-thirds of children with chronic asthma have symptoms that last well into adolescence [4]. Moreover, asthma is more common in girls than boys when it initially appears in puberty [5]. There is evidence that asthma in adolescents is underdiagnosed, with estimates showing that 20–30% of all asthma cases in this age group go undetected [2].

Due to their developmental stage, physiological changes, and the advent of psychosocial issues that might impact care, adolescents with asthma present a unique set of challenges for the practicing clinician. Poor asthma control may alter adolescents’ behavior and, more worryingly, a failure to recognize their symptoms is associated with poor treatment adherence. Noncompliance with asthma treatments is a well-known issue in the adolescence age group and is exacerbated by risky behaviors, including smoking, vaping, and other substance use, leading to unanticipated exacerbations and consequences. In Australia, electronic cigarette (e-cigarette) usage is becoming more popular among youths, with roughly 5% of 18–24-year-olds reporting experience with e-cigarettes. This growing trend is alarming, especially among first-time smokers [6,7].

Electronic cigarettes (ECs) and other electronic nicotine delivery systems (ENDSs) are now two to three times more popular among adolescents and young adults than among older adults, even though they were originally marketed as a form of smoking cessation assistance for adults with long-term cigarette use [8,9].

This review aims to present the current knowledge on the asthma–vaping interaction during the sentimentally fragile adolescence period, emphasizing the need for more well-designed studies that will prove causality and exert pressure on governments to restrict EC use by youths.

## 2. What Are ECs and Why Have They Become So Popular among Adolescents?

In 2003, the Chinese pharmacist Hon Lik produced and started marketing the first EC, which was introduced to the Chinese market a year later with little commercial success. A few years later, when the product was launched in Western markets, its popularity exceeded all expectations, with a current turnover of around 18.5 billion USD [10].

The main parts of a conventional vaping apparatus are a mouthpiece, an e-liquid- or e-juice-filled cartridge, a battery, and a heating component. When the device is turned on, the battery warms the heating component, which converts the e-liquids into an aerosol vapor that is inhaled. Inhaling and exhaling this aerosol is called vaping [11,12].

Since the introduction of vaping devices in Western markets, they have gained extreme popularity, especially among adolescents [13,14,15,16]. Multiple factors have contributed to this surge in vaping, with the most common being their flavoring, age-related curiosity, the perception that these products are “cool”, and the fact they are a healthier alternative compared to combustible tobacco products [17]. Moreover, smart advertising on behalf of the selling companies has augmented the vaping trend among adolescents. The marketing themes used are focused on the sense of freedom and rebellion and the glamor around vaping, along with dubious messages that ECs are both healthy and the best way to achieve tobacco cessation [18,19]. The main advertisement conduits have been radio, television, and social media. These products can be purchased through analogous websites, leading to the fast and wide adaptation of their habitual use among youths [20,21].

## 3. Are ECs a Means to Avoid or Quit Tobacco Smoking?

In contrast to EC advocates’ common argument that vaping may serve as a reduction tool for active tobacco smokers [22], public health services consider vaping as a bridge to the future use of combustible tobacco products [23]. A systematic review and meta-analysis that included more than 8000 adolescents and young adults assessed the initial use of ECs and the subsequent initiation of combustible cigarette smoking. It showed that EC use is associated with a pooled odds ratio of 3.50 for subsequent cigarette smoking initiation compared to non-users [24]. Another study showed that although EC use is associated with future combustible tobacco use, the opposite trend was not detected in the long term [25]. Moreover, data revealed an association between EC vaping and progression to more frequent and heavier patterns of cigarette smoking in adolescents six months later [26].

## 4. Is the Content of ECs Safe to Inhale?

ECs are marketed as devices that imitate regular cigarettes and deliver nicotine without other toxicants, or at least contain a lesser amount of them. The included e-liquids are made up of a stabilizing humectant, such as propylene glycol (PG) and/or vegetable glycerin (also known as glycerol) (VG), and one or more flavoring additives that are used to give the vape a distinct and attractive aroma; for example, they can mimic the flavor of tobacco or make the vape taste like foods (fruits, candies, or sweets) or drinks (coffee or alcoholic beverages) [27]. Studies have shown that although ECs contain fewer toxic substances compared to combustible cigarettes [28,29], users are still exposed to a variety of potentially harmful materials besides nicotine. There is still a lack of knowledge as to the real contents of these products and the nature of their by-products after heating. When 12 brands of EC were tested regarding their contents, detectable amounts of known carcinogens and toxic chemicals were found. Though the detectable levels were lower when compared to those of regular cigarettes, their presence confutes the myth that ECs deliver nicotine without added toxic substances [29].

An analysis of samples taken from the refilling dispenser of 56 e-cigarette devices revealed the presence of toxic metals (lead, Pb; chromium, Cr; and nickel, Ni) and metals that are toxic when inhaled (manganese, Mn; zinc, Zn). The metals were derived from the heating element and contaminated the e-liquid [30].

Discrepancies were also found between the declared amount and the actual amount of nicotine delivered per puff in three different EC cartridges. Notably, analyses showed the presence of a very low amount of nicotine even in EC cartridges listed as containing no nicotine [31].

A recent study added another potential harm to the expansive list of hidden hazards related to vaping. The study found bacterial (27%) and fungal (81%) organic contaminants in the single-use and refillable e-cigarette products of a great number of different manufacturers [32].

## 5. Potential Effect of Vaping on Airway Inflammation, Lung Function, and the Clinical Features of Asthmatic Adolescences

The effects of vaping components on human airway cells could be derived from three potential sources (apart from nicotine): the stabilizing humectant of the device (PG and VG), the flavorings, and the vaping by-products such as the aforementioned metals. PG, VG, and EC flavorings are all considered by the FDA as “Generally Recognized as Safe” (GRAS) and are allowed for consumption as food additives. Their effect on human airways through vaping is still dubious and under study [33,34]. Heated PG and VG are used in fog production for theatrical and entertainment purposes, and fog exposure is associated with the acute development of a cough and throat dryness. Long-term exposure has been linked with more severe respiratory complaints such as chest tightness, wheezing, and reduced lung function [35]. Flavoring chemicals that provide a clove or cinnamon aroma to the vapor are known skin irritants and potential generators of asthma when inhaled [27,36]. Apart from metals, the vapor consists of volatile organic compounds such as benzene, toluene, and nitrosamines, which are considered toxic agents [28,29,37].

Studies trying to clarify the effect of the vape on the asthmatic airway are lacking, especially when it comes to adolescents. Animal studies have consistently shown an increase in airway hyperresponsiveness on exposure to ECs. Researchers have also noticed a significant increase in airway inflammation (mainly Th2-dependent), characterized by an increased infiltration of eosinophils into the airway from blood. This is associated with an increase in mucus production and airway wall thickening. Th2-type inflammation has been confirmed by the detection of increased Th2 cytokine production, such as IL-4, IL-5, and IL-13, along with the presence of allergen-specific Inge [38,39,40,41].

Studies on asthmatic humans using ECs are characterized by two things: paucity in number and contradictory results. To the best of our knowledge, there are no studies on asthmatic adolescents’ lung function, and all data are derived from adult studies. Three interventional studies using impulse oscillometry (IOS) and conventional spirometry found an acute deterioration of IOS parameters after just one session of EC use. Peak expiratory flow and the FEV1/FVC ratio also deteriorated, along with airway resistance and small airway function. Fractional exhaled nitric oxide and Th2 cytokines increased, as did IL-1β, TNF-α, IL-10, and ISO8. All of these studies used healthy controls for comparison [42,43,44].

EC use can also undermine the way the human airway defends itself against infectious agents. When attacked, the airway employs three basic mechanisms to maintain its integrity: mucociliary clearance, cough, and innate immunity. Vaping increases the risk of respiratory infections by downregulating these mechanisms, since it impairs the function of the cilia, reduces the sensitivity of cough receptors, impairs neutrophil and alveolar macrophage function, and modifies the expression of genes and molecules involved in immune reactions [45,46,47,48]. Although these studies were not performed on asthmatic airways, infections are a well-known trigger of asthma exacerbations [49], and vaping may act as a mediator. There is a positive relationship between vaping and an enhanced risk of asthma development according to studies performed in children up to the age of 18 years. EC use increases the probability of an asthma diagnosis in adolescents [50,51,52] and has been found to be an independent risk factor for asthma, after controlling for various covariates [53,54,55]. Vaping also increases the asthma prevalence among high school students [56,57], and, according to an opinion article, the culprit may be the airway irritants and toxicants contained in EC flavorings and aerosols [58]. Cho et al., in a cross-sectional web-based study, found that adolescent EC users had a greater risk for school absenteeism due to asthma (high adjusted odds ratio for severe asthma) [50]. Interestingly, another school-based cross-sectional study investigating the effects of secondhand EC exposure on asthma showed that asthmatic youths have higher odds of reporting an asthma attack in the previous year when exposed to secondhand EC vapor [59].

Additionally, in two case reports, primary and secondhand exposure to ENDS in asthmatic adolescents resulted in severe asthma attacks and hypercarbic respiratory failure, necessitating the therapeutic application of veno-venous extracorporeal membrane oxygenation to overcome the attack [60]. This is in accordance with a previous study [59], suggesting that ENDS exposure, either primary or secondary, may cause lung function dysregulation and promote the onset of a severe asthma attack. Secondhand exposure to ENDS is also linked to respiratory symptomatology, airway smooth muscle constriction, and lung-health decline [61].

Han et al. showed that EC affects asthma prognosis and the use of an electronic vapor product is linked with lifelong asthma [62]. Four other studies revealed that among youths, asthmatics are more likely to be current vapers compared to non-asthmatics [54,63,64], and one of these studies emphasized that asthmatic adolescents using ECs have a much higher incidence of mental health problems such as depression and suicidality when compared to non-users [63].

Electronic vapor acute lung injury (EVALI) is a respiratory disease that presents (sub-) acutely with a range of clinical and pathological findings. The Centers for Disease Control and Prevention (CDC) has set diagnostic criteria for EVALI and has emphasized that the use of ECs in the previous 90 days before symptom initiation is mandatory, among other prerequisites [65]. An EVALI and asthma connection has been reported in several studies [66,67,68], and asthma has been found not only to be more common in patients hospitalized with EVALI (30–43.6% in adolescent asthmatics) when compared to the general population [69,70], but also as a factor associated with more severe outcomes in EVALI patients. A greater number of fatal cases of EVALI have a history of asthma (23%) compared to non-fatal cases (8%) [71].

**Figure 1:**
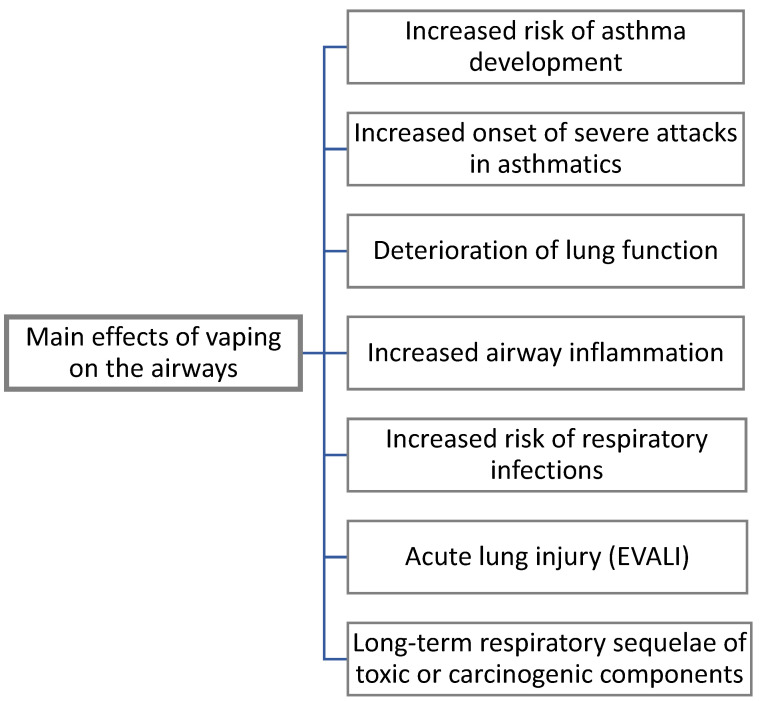
Evidenced effects of vaping on the airways.

EC exposure might seem less harmful compared to conventional tobacco smoke, but the evidence is derived from studies performed on specific amounts of vapor, that is, produced in a standardized way, which is far from what happens in real-life situations. An EC user’s exposure varies depending on the operating voltage of the device; the e-liquid temperature; and, finally, the user’s vaping habits [33]. For example, a study showed a 50-fold variation in the detected levels of nicotine in the aerosol produced by EC puffs when different voltages were used and alterations in length and speed were applied [34]. This means that the exact same e-liquid “behaves” differently when used in different EC settings and, moreover, that the smoking habits of the user can dramatically alter the type of exposure. As a result, when the effect of vaping on human health is investigated, the conclusions of any study may be not representative of what happens in real-life exposures (Figure 1).

## 6. What Can Be Done to Stop the EC Use Pandemic among Adolescents (Asthmatics or Not)?

Behind the EC use among adolescents, there is a growing industry that is highly profitable. Actions are needed at different levels to curb the threat rising from vaping. Several other reviews investigating the adverse effects of electronic cigarettes/vaping on adolescents’ health supported the need for urgent anti-vaping measures [72,73]. New, well-designed, and longitudinal studies are needed to substantiate the negative effects of vaping on young asthmatics and to convince governments to enact laws against this deleterious habit. Delivery systems that contain nicotine must be considered tobacco products; adolescents must not be allowed to purchase EC products; and flavors should not be permitted to be added in electronic nicotine products, since this “beautifies” their taste and renders them more attractive. Finally, their advertisement on all social media channels must be under government control and adolescent access must be restricted. The medical community and health authorities should remain vigilant, and the long delay before the devastating health effects of tobacco products were realized and counteracted in the past must not be forgotten or repeated.

## 7. Conclusions

Although studies have highlighted the potential adverse effect of EC use on asthma, most of them are cross-sectional studies, limited by their design, and they do not allow us to establish a clear cause-and-effect relationship between vape exposure and asthma symptoms. However, they contain epidemiological data that reflect the current habits of adolescents and may provide a means for early and effective interference. Well-designed prospective cohort studies aiming to understand the relationship between lung morbidity and EC use in adolescents are urgently needed. Nevertheless, studies have shown that ECs contain potentially toxic components such as PG, VG, varying amounts of nicotine, and flavoring chemicals that may induce respiratory morbidity, especially on the inflamed, asthmatic airway. Markedly, these adverse effects are not similar to those caused by combustible cigarettes, but this dissimilarity does not mean that EC use is “healthier” or preferable to conventional tobacco products. There has been a long list of continually evolving e-cigarette devices since their introduction, and all of them, irrespective of their construction (including heated tobacco units, HEETS, and pods) are undoubtedly a health threat, especially for children and adolescents. Actions to curb EC use are needed without delay, and they must be effective and scientifically supported. For this purpose, we need to keep our medical evidence-based data constantly updated in order to keep pace with the swiftly evolving EC industry.

## References

[1] United Nations General Assembly (1989). Convention on the Rights of the Child, Treaty Series.

[2] British Thoracic Society, Scottish Intercollegiate Guidelines Network (2019). British Guideline on the Management of Asthma. https://www.sign.ac.uk/media/1048/sign158.pdf.

[3] Australian Institute of Health and Welfare (2019). Asthma. https://www.aihw.gov.au/reports/chronic-respiratory-conditions/asthma.

[4] Nicolai T., Illi S., Tenbörg J., Kiess W., Mutius E.V. (2001). Puberty and prognosis of asthma and bronchial hyper-reactivity. Pediatric Allergy Immunol..

[5] Towns S.J., van Asperen P.P. (2009). Diagnosis and management of asthma in adolescents. Clin. Respir. J..

[6] Australian Institute of Health and Welfare (2020). Alcohol, Tobacco and Other Drugs in Australia. https://www.aihw.gov.au/reports/alcohol/alcohol-tobacco-other-drugs-australia.

[7] Thanavala Y., Goniewicz M.L. (2019). Vaping-induced severe respiratory disease outbreak: What went wrong?. Lancet Respir. Med..

[8] Jamal A., Gentzke A., Hu S.S., Cullen K.A., Apelberg B.J., Homa D.M., King B.A. (2017). Tobacco Use among Middle and High School Students—United States, 2011–2016. MMWR Morb. Mortal. Wkly. Rep..

[9] (2016). QuickStats: Cigarette Smoking Status Among Current Adult E-cigarette Users, by Age Group—National Health Interview Survey, United States, 2015. MMWR Morb. Mortal. Wkly. Rep..

[10] US Department of Health and Human Services (2016). E-Cigarette Use Among Youth and Young Adults: A Report of the Surgeon General. https://e-cigarettes.surgeongeneral.gov/documents/2016_SGR_Full_Report_non-508.pdf.

[11] Blagev D.P., Harris D., Dunn A.C., Guidry D.W., Grissom C.K., Lanspa M.J. (2019). Clinical presentation, treatment, and short-term outcomes of lung injury associated with e-cigarettes or vaping: A prospective observational cohort study. Lancet.

[12] Alexander L., Perez M.F. (2019). Identifying, tracking, and treating lung injury associated with e-cigarettes or vaping. Lancet.

[13] Camenga D.R., Kong G., Cavallo D.A., Liss A., Hyland A., Delmerico J., Cummings K.M., Krishnan-Sarin S. (2014). Alternate tobacco product and drug use among adolescents who use electronic cigarettes, cigarettes only, and never smokers. J. Adolesc. Health.

[14] Centers for Disease Control (2013). Electronic cigarette use among middle and high school students—United States, 2011–2012. Morb. Mortal. Wkly. Rep..

[15] Dutra L.M., Glantz S.A. (2014). Electronic cigarettes and conventional cigarette use among us adolescents: A cross-sectional study. JAMA Pediatrics.

[16] Lee S., Kimm H., Yun J.E., Jee S.H. (2011). Public health challenges of electronic cigarettes in South Korea. J. Prev. Med. Public Health.

[17] Kong G., Morean M.E., Cavallo D.A., Camenga D.R., Krishnan-Sarin S. (2015). Reasons for Electronic Cigarette Experimentation and Discontinuation Among Adolescents and Young Adults. Nicotine Tob. Res..

[18] Grana R.A., Ling P.M. (2014). “Smoking Revolution”: A Content Analysis of Electronic Cigarette Retail Websites. Am. J. Prev. Med..

[19] Zhu S.H., Sun J.Y., Bonnevie E., Cummins S.E., Gamst A., Yin L., Lee M. (2014). Four hundred and sixty brands of e-cigarettes and counting: Implications for product regulation. Tob. Control.

[20] Zhu S., Gamst A., Lee M., Cummins S., Yin L., Zoref L. (2013). The use and perception of electronic cigarettes and snus among the U.S. population. PLoS ONE.

[21] Jones K., Salzman G. (2020). The Vaping Epidemic in Adolescents. Mo Med..

[22] Cahn Z., Siegel M. (2011). Electronic cigarettes as a harm reduction strategy for tobacco control: A step forward or a repeat of past mistakes?. J. Public Health Policy.

[23] Riker C.A., Lee K., Darville A., Hahn E.J. (2012). E-cigarettes: Promise or peril?. Nurs. Clin. N. Am..

[24] Soneji S., Barrington-Trimis J.L., Wills T.A., Leventhal A.M., Unger J.B., Gibson L.A., Sargent J.D. (2017). Association Between Initial Use of e-Cigarettes and Subsequent Cigarette Smoking Among Adolescents and Young Adults: A Systematic Review and Meta-analysis. JAMA Pediatrics.

[25] Bold K.W., Kong G., Camenga D.R., Simon P., Cavallo D.A., Morean M.E., Krishnan-Sarin S. (2018). Trajectories of E-Cigarette and Conventional Cigarette Use Among Youth. Pediatrics.

[26] Leventhal A.M., Stone M.D., Andrabi N., Barrington-Trimis J., Strong D.R., Sussman S., Audrain-McGovern J. (2016). Association of e-Cigarette Vaping and Progression to Heavier Patterns of Cigarette Smoking. JAMA.

[27] Clapp P.W., Jaspers I. (2017). Electronic Cigarettes: Their Constituents and Potential Links to Asthma. Curr. Allergy Asthma Rep..

[28] Williams M., Villarreal A., Bozhilov K., Lin S., Talbot P. (2013). Metal and Silicate Particles Including Nanoparticles Are Present in Electronic Cigarette Cartomizer Fluid and Aerosol. PLoS ONE.

[29] Goniewicz M.L., Knysak J., Gawron M., Kosmider L., Sobczak A., Kurek J., Prokopowicz A., Jablonska-Czapla M., Rosik-Dulewska C., Havel C. (2014). Levels of selected carcinogens and toxicants in vapor from electronic cigarettes. Tob. Control.

[30] Olmedo P., Goessler W., Tanda S., Grau-Perez M., Jarmul S., Aherrera A., Chen R., Hilpert M., Cohen J.E., Navas-Acien A. (2018). Metal Concentrations in e-Cigarette Liquid and Aerosol Samples: The Contribution of Metallic Coils. Environ. Health Perspect..

[31] Trehy M.L., Ye W., Hadwiger M.E., Moore T.W., Allgire J.F., Woodruff J.T., Ahadi S.S., Black J.C., Westenberger B.J. (2011). Analysis of electronic cigarette cartridges, refill solutions, and smoke for nicotine and nicotine related impurities. J. Liq. Chromatogr. Relat. Technol..

[32] Schmidt S. (2019). Microbial Toxins in E-Liquid: A Potential New Vaping-Related Exposure to Explore. Environ. Health Perspect..

[33] Kaur G., Pinkston R., Mclemore B., Dorsey W.C., Batra S. (2018). Immunological and toxicological risk assessment of e-cigarettes. Eur. Respir. Rev..

[34] Talih S., Balhas Z., Eissenberg T., Salman R., Karaoghlanian N., El Hellani A., Baalbaki R., Saliba N., Shihadeh A. (2015). Effects of user puff topography, device voltage, and liquid nicotine concentration on electronic cigarette nicotine yield: Measurements and model predictions. Nicotine Tob. Res..

[35] Varughese S., Teschke K., Brauer M., Chow Y., Van Netten C., Kennedy S.M. (2005). Effects of theatrical smokes and fogs on respiratory health in the entertainment industry. Am. J. Ind. Med..

[36] López Sáez M.P., Carrillo P., Huertas A.J., Fernández Nieto M., Lopez J.D. (2015). Occupational asthma and dermatitis induced by eugenol in a cleaner. J. Investig. Allergol. Clin. Immunol..

[37] Rubinstein M.L., Delucchi K., Benowitz N.L., Ramo D.E. (2018). Adolescent exposure to toxic volatile Organic chemicals from e-cigarettes. Pediatrics.

[38] Marczylo T. (2020). How bad are e-cigarettes? What can we learn from animal exposure models?. J. Physiol..

[39] McAlinden K.D., Naidu V., Sohal S.S., Sharma P. (2020). In utero Exposure to Nicotine Containing Electronic Cigarettes Increases the Risk of Allergic Asthma in Female Offspring. Am. J. Physiol. Lung Cell Mol. Physiol..

[40] Chapman D.G., Casey D.T., Ather J.L., Aliyeva M., Daphtary N., Lahue K.G., van der Velden J.L., Janssen-Heininger Y.M.W., Irvin C.G. (2019). The Effect of Flavored E-cigarettes on Murine Allergic Airways Disease. Sci. Rep..

[41] Lim H.B., Kim S.H. (2014). Inhallation of e-cigarette Cartridge Solution Aggravates Allergen-induced Airway Inflammation and Hyper-responsiveness in Mice. Toxicol. Res..

[42] Lappas A.S., Tzortzi A.S., Konstantinidi E.M., Teloniatis S.I., Tzavara C.K., Gennimata S.A., Koulouris N.G., Behrakis P.K. (2018). Short-term respiratory effects of e-cigarettes in healthy individuals and smokers with asthma. Respirology.

[43] Palamidas A., Tsikrika S., Katsaounou P.A., Vakali S., Gennimata S.A., Kaltsakas G., Gratziou C., Koulouris N. (2017). Acute effects of short-term use of e-cigarettes on airways physiology and respiratory symptoms in smokers with and without airway obstructive diseases and in healthy non-smokers. Tob. Prev. Cessat..

[44] Kotoulas S.C., Pataka A., Domvri K., Spyratos D., Katsaounou P., Porpodis K., Fouka E., Markopoulou A., Passa-Fekete K., Grigoriou I. (2020). Acute effects of e-cigarette vaping on pulmonary function and airway inflammation in healthy individuals and in patients with asthma. Respirology.

[45] Dicpinigaitis P.V., Chang A.L., Dicpinigaitis A.J., Negassa A. (2016). Effect of e-Cigarette Use on Cough Reflex Sensitivity. Chest.

[46] Sussan T.E., Gajghate S., Thimmulappa R.K., Ma J., Kim J.H., Sudini K., Consolini N., Cormier S.A., Lomnicki S., Hasan F. (2015). Exposure to electronic cigarettes impairs pulmonary anti-bacterial and anti-viral defenses in a mouse model. PLoS ONE.

[47] Clapp P.W., Pawlak E.A., Lackey J.T., Keating J.E., Reeber S.L., Glish G.L., Jaspers I. (2017). Flavored e-cigarette liquids and cinnamaldehyde impair respiratory innate immune cell function. Am. J. Physiol. Lung Cell. Mol. Physiol..

[48] Martin E.M., Clapp P.W., Rebuli M.E., Pawlak E.A., Glista-Baker E., Benowitz N.L., Fry R.C., Jaspers I. (2016). E-cigarette use results in suppression of immune and inflammatory-response genes in nasal epithelial cells similar to cigarette smoke. Am. J. Physiol. Lung Cell. Mol. Physiol..

[49] Busse W.W., Lemanske R.F., Gern J.E. (2010). Role of viral respiratory infections in asthma and asthma exacerbations. Lancet.

[50] Cho J.H., Paik S.Y. (2016). Association between Electronic Cigarette Use and Asthma among High School Students in South Korea. PLoS ONE.

[51] Chung S.J., Kim B.K., Oh J.H., Shim J.S., Chang Y.S., Cho S.H., Yang M.S. (2020). Novel tobacco products including electronic cigarette and heated tobacco products increase risk of allergic rhinitis and asthma in adolescents: Analysis of Korean youth survey. Allergy.

[52] Kim S.Y., Sim S., Choi H.G. (2017). Active, passive, and electronic cigarette smoking is associated with asthma in adolescents. Sci. Rep..

[53] Schweitzer R.J., Wills T.A., Tam E., Pagano I., Choi K. (2017). E-cigarette use and asthma in a multiethnic sample of adolescents. Prev. Med..

[54] Fedele D.A., Barnett T.E., Dekevich D., Gibson-Young L.M., Martinasek M., Jagger M.A. (2016). Prevalence of and beliefs about electronic cigarettes and hookah among high school students with asthma. Ann. Epidemiol..

[55] Wills T.A., Choi K., Pagano I. (2020). E-Cigarette Use Associated With Asthma Independent of Cigarette Smoking and Marijuana in a 2017 National Sample of Adolescents. J. Adolesc. Health.

[56] Xie L., Rao D.R., Harrell M.B., Vidot D.C., Gelfand A., Sterling K., Messiah S.E. (2020). Ethnic disparities in the e-cigarette use epidemic and childhood asthma in the US. Pediatr. Pulmonol..

[57] Alnajem A., Redha A., Alroumi D., Alshammasi A., Ali M., Alhussaini M., Almutairi W., Esmaeil A., Ziyab A.H. (2020). Use of electronic cigarettes and secondhand exposure to their aerosols are associated with asthma symptoms among adolescents: A cross-sectional study. Respir. Res..

[58] Galderisi A., Ferraro V.A., Caserotti M., Quareni L., Perilongo G., Baraldi E. (2020). Protecting youth from the vaping epidemic. Pediatr. Allergy Immunol..

[59] Bayly J.E., Bernat D., Porter L., Choi K. (2019). Secondhand Exposure to Aerosols from Electronic Nicotine Delivery Systems and Asthma Exacerbations Among Youth with Asthma. Chest.

[60] Bradford L.E., Rebuli M.E., Ring B.J., Jaspers I., Clement K.C., Loughlin C.E. (2020). Danger in the vapor? ECMO for adolescents with status asthmaticus after vaping. J. Asthma.

[61] Di Cicco M., Sepich M., Ragazzo V., Peroni D.G., Comberiati P. (2020). Potential effects of e-cigarettes and vaping on pediatric asthma. Minerva Pediatr..

[62] Han Y.Y., Rosser F., Forno E., Celedón J.C. (2020). Electronic vapor products, marijuana use, smoking, and asthma in US adolescents. J. Allergy Clin. Immunol..

[63] Kim C.W., Jeong S.C., Kim J.Y., Lee J.S., Lee J.H., Jo S.H., Kim S.H. (2020). Associated factors for depression, suicidal ideation and suicide attempt among asthmatic adolescents with experience of electronic cigarette use. Tob. Induc. Dis..

[64] Larsen K., Faulkner G.E.J., Boak A., Hamilton H.A., Mann R.E., Irving H.M., To T. (2016). Canadian Respiratory Research Network. Looking beyond cigarettes: Are Ontario adolescents with asthma less likely to smoke e-cigarettes, marijuana, waterpipes or tobacco cigarettes?. Respir. Med..

[65] Schier J.G., Meiman J.G., Layden J., Mikosz C.A., VanFrank B., King B.A., Salvatore P.P., Weissman D.N., Thomas J., Melstrom P.C. (2019). Severe Pulmonary Disease Associated with Electronic-Cigarette-Product Use—Interim Guidance. MMWR Morb. Mortal. Wkly. Rep..

[66] Aberegg S.K., Maddock S.D., Blagev D.P., Callahan S.J. (2020). Diagnosis of EVALI: General Approach and the Role of Bronchoscopy. Chest.

[67] Rodriguez J.A., Roa A.A., Lemos-Ramirez J.C. (2020). E-Cigarette or Vaping Product Use-Associated Lung Injury (EVALI) Mimicking COVID-19 Disease. Case Rep. Pulmonol..

[68] Chawla H., Weiler T.E. (2020). Cigarette or Vaping Product Use-Associated Lung Injury Presenting as Sub-Acute Hypoxemia Without Increased Work of Breathing. Cureus.

[69] Clapp P.W., Peden D.B., Jaspers I. (2020). E-cigarettes, vaping related pulmonary illnesses, and asthma: A perspective from inhalation toxicologists. J. Allergy Clin. Immunol..

[70] Adkins S.H., Anderson K.N., Goodman A.B., Twentyman E., Danielson M.L., Kimball A., Click E.S., Ko J.Y., Evans M.E., Weissman D.N. (2020). Demographics, Substance Use Behaviors, and Clinical Characteristics Adolescents with e-Cigarette, or Vaping, Product Use-Associated Lung Injury (EVALI) in the United States in 2019. JAMA Pediatrics.

[71] Werner A.K., Koumans E.H., Chatham Stephens K., Salvatore P.P., Armatas C., Byers P., Clark C.R., Ghinai I., Holzbauer S.M., Navarette K.A. (2020). Hospitalizations and Deaths Associated with EVALI. N. Engl. J. Med..

[72] Xian S., Chen Y. (2021). E-cigarette users have associated with asthma disease: A meta-analysis. Clin. Respir. J..

[73] Miyashita L., Foley G. (2020). E-cigarettes and respiratory health: The latest evidence. J. Physiol..

